# Aspiration versus peritoneal lavage in appendicitis: a meta-analysis

**DOI:** 10.1186/s13017-021-00391-y

**Published:** 2021-09-06

**Authors:** Gloria Burini, Maria Chiara Cianci, Marco Coccetta, Alessandro Spizzirri, Salomone Di Saverio, Riccardo Coletta, Paolo Sapienza, Andrea Mingoli, Roberto Cirocchi, Antonino Morabito

**Affiliations:** 1 General and Emergency Surgical Clinic of Ancona, Ancona, Italy; 2 Department of Pediatric Surgery, Meyer Children’s Hospital, University of Florence, Florence, Italy; 3 Hospital of Terni, Terni, Italy; 4 Department of Colorectal Surgery, Addenbrooke’s Hospital, Cambridge University Hospitals NHS Foundation Trust, Hills Road, Cambridge, CB2 0QQ UK; 5 Department of Pediatric Surgery, Meyer Children’s Hospital, School of Environment and Life Science, University of Salford, Salford, UK; 6 Department of Surgery, University of Rome, Sapienza, Italy; 7 Department of Medicine and Surgery, University of Perugia, Perugia, Italy; 8 Department of Pediatric Surgery, Meyer Children’s Hospital, Department of Neurofarba, University of Florence, Florence, Italy

**Keywords:** Complicated appendicitis, Post-operative intra-abdominal abscess, Peritoneal irrigation, Abscess suction

## Abstract

**Background:**

Acute appendicitis is one of the most frequent abdominal surgical emergencies. Intra-abdominal abscess is a frequent post-operative complication. The aim of this meta-analysis was to compare peritoneal irrigation and suction versus suction only when performing appendectomy for complicated appendicitis.

**Methods:**

According to PRISMA guidelines, a systematic review was conducted and registered into the Prospero register (CRD42020186848). The risk of bias was defined to be from low to moderate.

**Results:**

Seventeen studies (9 RCTs and 8 CCTs) were selected, including 5315 patients. There was no statistical significance in post-operative intra-abdominal abscess in open (RR 1.27, 95% CI 0.75–2.15; *I*^2^ = 74%) and laparoscopic group (RR 1.51, 95% CI 0.73–3.13; *I*^2^ = 83%). No statistical significance in reoperation rate in open (RR 1.27, 95% CI 0.04–2.49; *I*^2^ = 18%) and laparoscopic group (RR 1.42, 95% CI 0.64–2.49; *I*^2^ = 18%). In both open and laparoscopic groups, operative time was lower in the suction group (RR 7.13, 95% CI 3.14–11.12); no statistical significance was found for hospital stay (MD − 0.39, 95% CI − 1.07 to 0.30; *I*^2^ = 91%) and the rate of wound infection (MD 1.16, 95% CI 0.56–2.38; *I*^2^ = 71%).

**Conclusions:**

This systematic review has failed to demonstrate the statistical superiority of employing intra-operative peritoneal irrigation and suction over suction-only to reduce the rate of post-operative complications after appendectomy, but all the articles report clinical superiority in terms of post-operative abscess, wound infection and operative times in suction-only group.

**Supplementary Information:**

The online version contains supplementary material available at 10.1186/s13017-021-00391-y.

## Background

Acute appendicitis is one of the most frequent abdominal surgical emergencies. It occurs with a rate of 8.6% for males and 6.7% for females [1]. Approximately 8% of the population will undergo appendectomy for acute appendicitis in their lifetime [2]. Complicated appendicitis represents from 14 to 55% of all appendicitis cases and is defined as an acute appendicitis with peritonitis, rupture, gangrene or intra-abdominal abscess, intra-peritoneal presence of faecalith, intra-operative mass or four-quadrant purulent material, local or generalized peritonitis [3, 4]. Some studies have demonstrated that complicated appendicitis is associated with higher rates of major post-operative complications [5–8]. In children, perforated appendicitis has an incidence from 15 to 20% [7] and can be responsible for post-operative morbidity [1], especially related to post-operative IAA. In particular, perforated appendicitis leads more frequently to IAA [8–10], increasing the risk of surgical site infection, hospital stay and cost. The rate of IAA after appendectomy for perforated appendicitis is variable: some studies report between 5 and 10%, others 3–20% [9, 10]. Perforated appendicitis was recognized as an independent risk factor for development of post-operative IAA [7–9]. Different studies defined a risk of post-operative IAA of 1–4% in non-perforated appendicitis [7–9], compared with 10–24% after appendectomy for perforated appendicitis [9, 10]. Diabetes mellitus, young and old age [6], obesity and peritoneal irrigation were associated risk factors [6, 8].

Laparoscopic appendectomy has become the standard surgical procedure for acute appendicitis in both the paediatric and adult population [6, 11–13]. Recognized benefits are a more rapid recovery, less pain, fewer wound infections, shorter hospitalization and earlier return to daily activities [6, 11, 14, 15]. Although appendectomy is considered a safe procedure, different post-operative complications may occur, such as intra-abdominal abscess (IAA), wound infection, bleeding and bowel obstruction.

Most frequent and fearsome complication is abscess that occurs in 2.2% after laparoscopic appendectomy [16], but in both laparoscopic and open techniques, intra-abdominal abscess seems to be frequent, regardless of the technique. Its incidence is related especially to the severity of the appendicitis; patients with complicated appendicitis are more likely to develop an IAA regardless of technique [16, 17]. In the paediatric population, the incidence of post-operative intra-abdominal abscess after appendectomy for complicated/perforated appendicitis is approximately 24% [9]. Abscess development is most frequently observed in perforated appendicitis [7, 10], which represents the main risk factor for this complication to occur [7–9].

Post-operative IAA is associated with [1, 18] significant morbidity, patient discomfort, prolonged hospital stay and increased cost, often necessitating readmission and repeat intervention [7, 19–22].

The management of IAA remains controversial with different strategies suggested to decrease its incidence: antibiotic prophylaxis [23, 24], post-operative antibiotic therapy [25], peritoneal irrigation with saline solution [26] or suction only of the abscess/purulent liquid without irrigation of the cavity during appendectomy. In the literature, many studies address this topic; however, currently there is no evidence to clearly demonstrate the effectiveness of peritoneal irrigation over suction only. Italian guidelines recommend thorough peritoneal lavage (6–8 L of warm saline) and aspiration to minimize the IAA rate in complicated appendicitis [27]. The recent WSES (World Society of Emergency Surgery) guidelines report that “Peritoneal irrigation does not have any advantage over suction alone in complicated appendicitis in both adults and children. The performance of irrigation during laparoscopic appendectomy does not seem to prevent the development of IAA and wound infections in neither adults nor paediatric patients”. WSES recommendation is “to perform suction only in complicated appendicitis patients with intra-abdominal collections undergoing laparoscopic appendectomy” [QoE: Moderate; Strength of recommendation: Strong; 1B]) [28]. The concern regarding irrigation and lavage is that these procedures might help spread the infectious material [29]. This systematic review of the literature aims to evaluate the available data on comparative studies regarding peritoneal irrigation and suction vs suction only when performing appendectomy for complicated appendicitis.

## Main text

### Methods

This systematic review adheres to AMSTAR 2 criteria and examines the available data on comparative studies about irrigation and suction vs suction only during appendectomy [30].

The literature was searched using PubMed, Scopus and Web of Science up to 10 February 2021 including the following terms: “peritoneal irrigation” OR “peritoneal lavage” combined with “ruptured appendicitis” OR “perforated appendicitis”.

No language restrictions were enforced. All titles and abstracts were analysed to select the studies that reported intra-operative lavage during appendectomy. To avoid duplication of data, only the most recent review was considered in case of overlapping study periods for articles performed by the same research groups.

The Preferred Reporting Items for Systematic Reviews and Meta-analyses (PRISMA) guidelines were followed [31].

The PubMed function “related articles” was used to expand each search and the reference list of all potentially eligible studies was evaluated. Full texts were then independently screened by two authors (S.V. and R.C.), and a final consensus was reached by the same authors. No disagreements were reported.

For each study, the following data were extracted and summarized: author’s surname, year of publication, country of the hospital in which the procedures were performed, time of patient enrolment, study design, number of patients who underwent appendectomy, number of patients who underwent lavage and suction and suction only, age of patients, BMI, gender, severity of appendicitis and volume of peritoneal irrigation.

Primary outcomes were identified for both groups (irrigation and suction vs suction only) for laparoscopic and open appendectomy:

- Post-operative abdominal abscess.
- Reoperation for abdominal abscess.

The following data were considered as secondary outcomes:

- Duration of surgical treatment.
- Length of stay.
- Wound infections.

A subgroup analysis was also performed distinguishing the RCTs and CCTs (controlled clinical trials).

Assessment of methodological quality was performed independently by two authors. The risk of bias in the RCTs was assessed using methods described in the Cochrane Handbook for Systematic Reviews of Interventions [32] and the Risk of Bias In Non-randomized Studies of Interventions (ROBINS-I) assessment tool [33, 34]. Review questions and aims, outcomes, relevant confounding domains and co-interventions that could impact outcomes were assessed.

The systematic review and meta-analysis was registered into the Prospero international prospective register of systematic reviews (CRD42020186848).

Statistical analysis was performed with the Review Manager (RevMan version 5.3.5) computer program (Copenhagen: The Nordic Cochrane Centre, The Cochrane Collaboration, 2014).

Dichotomous outcomes were pooled with a random-effects model applying the Mantel–Haenszel method to estimate risk ratios (RR) and their 95% confidence intervals [35]. Clinical heterogeneity was tested using *τ*^2^, Cochrane’s Q and *I*^2^ statistics. An *I*^2^ value exceeding 50% was considered indicative for heterogeneity.

The random-effects analysis model was used for high clinical heterogeneity and statistically significant higher Chi-squared value and *I*^2^ [36]. In all remaining circumstances, the fixed-effects model was applied [37].

### Results

An initial literature search based on the evaluation of title and abstract yielded 841 reviews, and the results of searches are reported in Additional file 1: SDC searches.

**Fig. 1:**
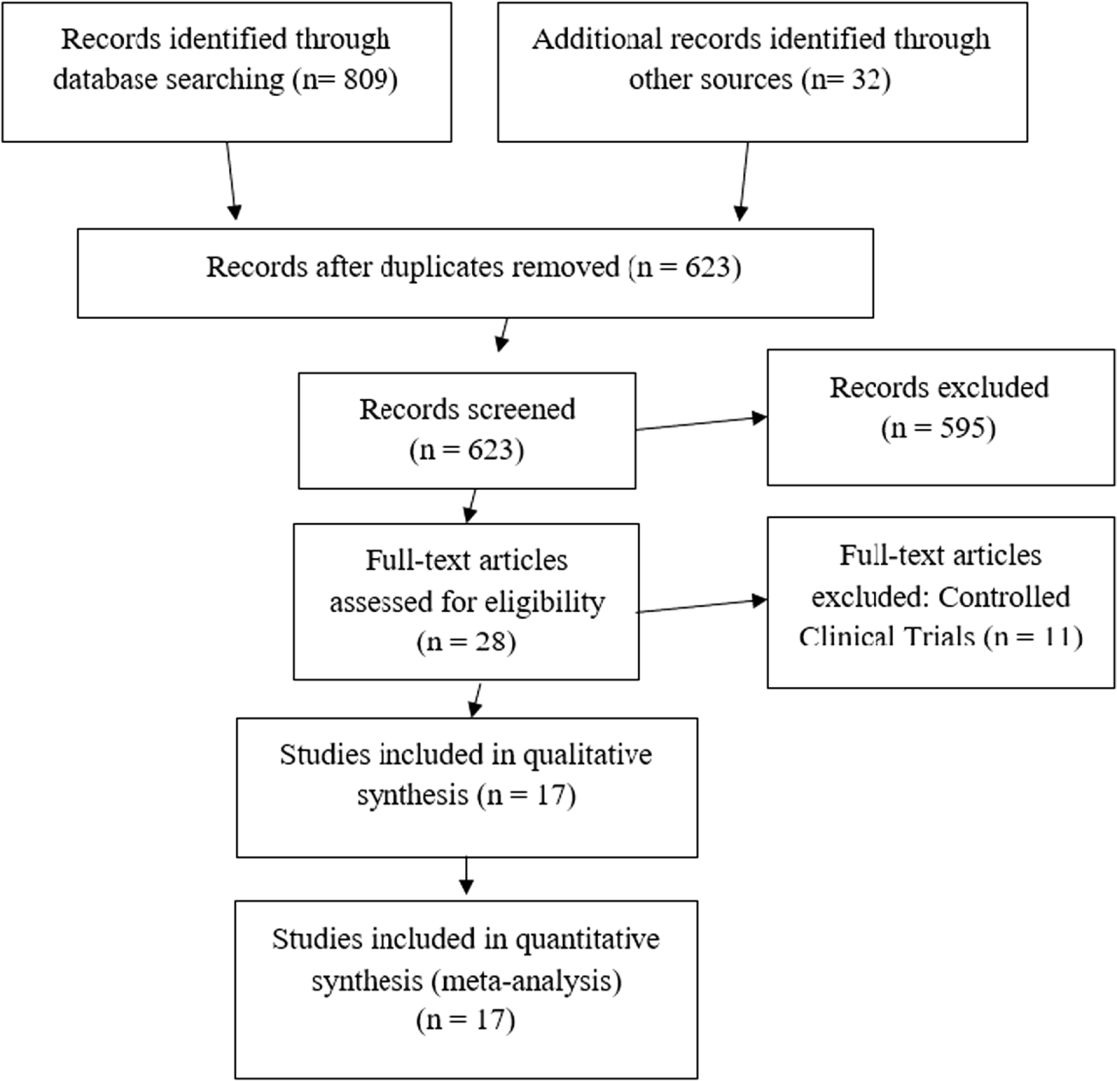
PRISMA flow diagram

Of them, 30 articles (including duplicated studies, case reports, articles without related data, review and meta-analysis publication) did not meet the inclusion criteria. The full text of 28 papers was reviewed: 11 studies were excluded (Additional file 2: Table S1) [19, 38–47] and 17 were selected for the meta-analysis [4, 6, 8, 10, 48–60], according to PRISMA flow diagram (Fig. 1).

The 17 selected studies (9 RCTs and 8 CCTs) included 5315 patients (2532 irrigation and suction versus 2783 irrigation) (Table 1).

**Table 1:** Characteristics of the RCT and observational studies included

Study	Nation	Type of study	Time of enrolment	Number of patients enrolled	Irrigation and suction	Suction
Gemici 2020	Turkey	RCT	2014–2017	286	112 (39.2%)	174 (60.8%)
Anderson 2020	Texas	RCT	2016–2017	100	50 (50%)	50 (50%)
Nataraja 2019	Australia, China	RCT	2015–2018	86	44 (51.2%)	42 (48.8%)
Lee 2019	Korea	CCT	2014–2016	553	207 (37.4%)	346 (62.6%)
Escolino 2018	Italy France, United Kingdom, Austria, Missouri	CCT	Over 5 years	699	488 (69.8%)	211 (30.2%)
Sardiwalla 2018	South Africa	RCT	2016–2017	86	42 (48.8%)	44 (51.2%)
Sun 2017	China	RCT	2015–2016	260	130 (50%)	130 (50%)
Snow 2016	Australia	RCT	2013–2015	81	40 (49.4%)	41 (50.6%)
Cho 2015	Korea	CCT	2010–2013	1817	731 (40.2%)	1086 (59.8%)
Hartwich 2013	USA	CCT	2005–2011	237	139 (58,6%)	98 (41,4%)
St. Peter 2012	USA	RCT	2008–2011	220	110 (50%)	110 (50%)
Akkoyun 2012	Turkey	CCT	1998–2011	234	61 (26.1%)	173 (73.9%)
Moore 2011	USA	CCT	2007–2008	176	115 (65.3%)	61 (34.7%)
Toki 1995	Japan	RCT	1984–1993	53	29 (54.7%)	24 (45.3%)
Buanes 1991	Norway	RCT	1984–1987	83	39 (47%)	44 (53%)
Stewart 1978	Great Britain	CCT	1965–1974	189	117 (61.9%)	72 (38.1%)
Normann 1975	Norway	CCT	1968–1973	155	78 (50.3%)	77 (49.7%)

*RCT* randomized controlled trial, *CCT* controlled clinical trial

All studies were published between 1975 and 2020 and written in English language. One review did not report patients’ age [6], eight studies were performed in paediatric patients and the other eight were performed in adults.

The average age in the washing and aspiration cohort ranged from 8.8 to 10.4 years for the paediatric subgroup and from 25.7 to 42 years for adults. In the suction alone cohort, instead, the relative average ages ranged from 9.1 to 10.9 years (paediatric patients) and from 20 to 44 years (adult subgroup). The characteristics of patients are reported in Additional file 3: SDC baseline.

### Quality assessment of RCTs

No data about random sequence generation were reported in 44.4% of the RCTs (four studies) and no data about allocation concealment in 33.3% of the RCTs (three studies).

A “low risk of bias” was reported in the analysis of the attrition bias, and an “unclear risk of bias” was identified for selective reporting (Additional file 4: SDC 3a, Additional file 5: SDC 3b). The risk of bias of random sequence generation and allocation concealment was low.

### Quality assessment of non-RCTs

According to ROBINS-I tool, risk-of-bias judgement may be identified as few, moderate, serious or critical. Three out of eight studies were assessed as low risk of overall bias, while five were determined to have a moderate risk. Regarding bias due to confounding, four out of eight studies were evaluated as having a low risk since authors used an appropriate analysis method that checked for all the relevant confounding domains. Furthermore, intervention discontinuations related to factors that are prognostic for the outcomes were not identified. On the other hand, four out of eight studies seemed to have a moderate risk of bias due to confounding (Additional file 6: SDC 4). Regarding bias in classification of the interventions, all studies had a low risk: intervention groups were clearly identified and they were not affected by knowledge of the outcome. Outcomes were clearly defined and measures were not influenced by knowledge of the intervention received. The methods of outcome assessment were comparable across intervention groups and no systematic errors were detected. Analysing bias due to missing data, three reviews were assessed as moderate risk.

## Results of effects of interventions

### Primary outcomes

#### Post-operative abdominal abscess after appendectomy

**Fig. 2:**
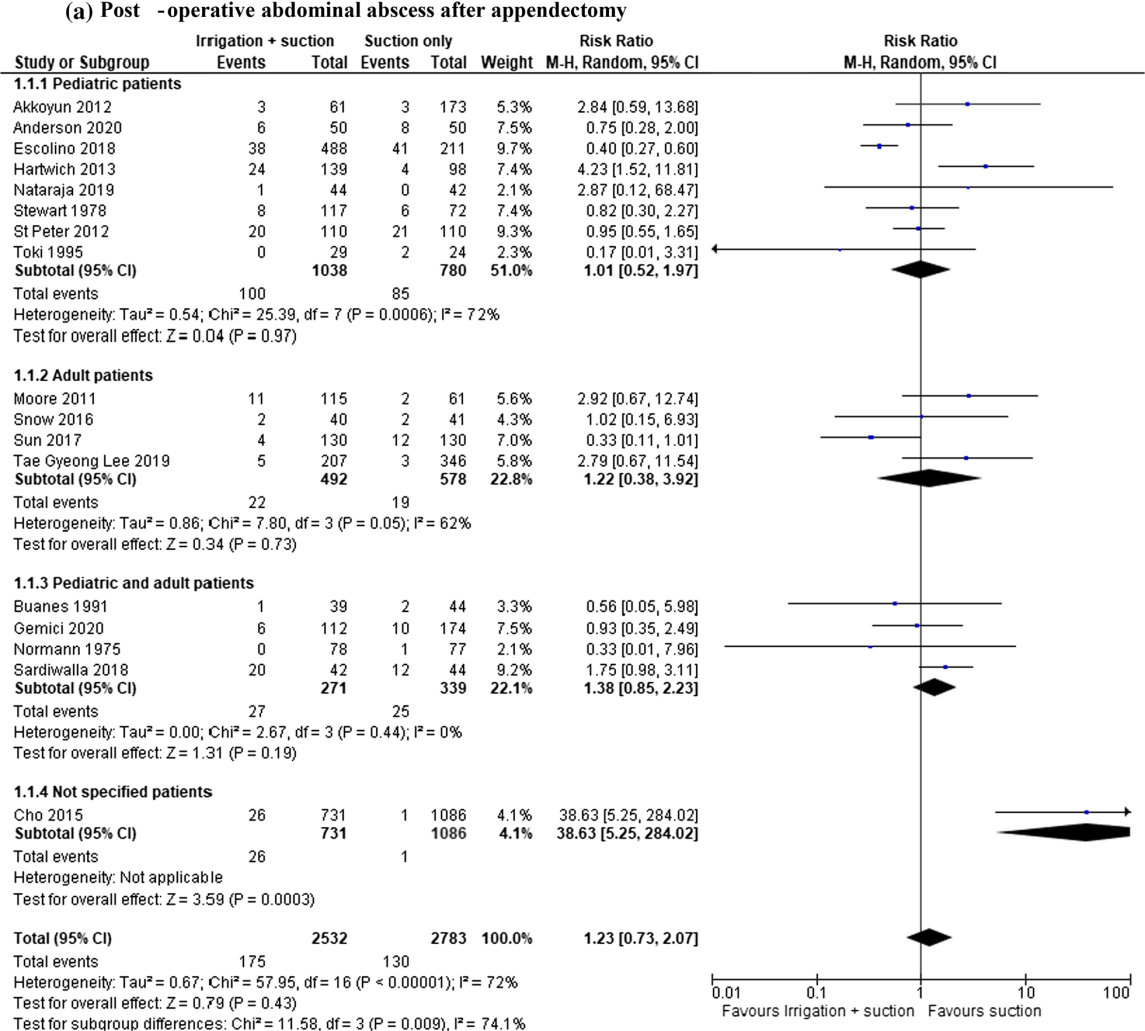

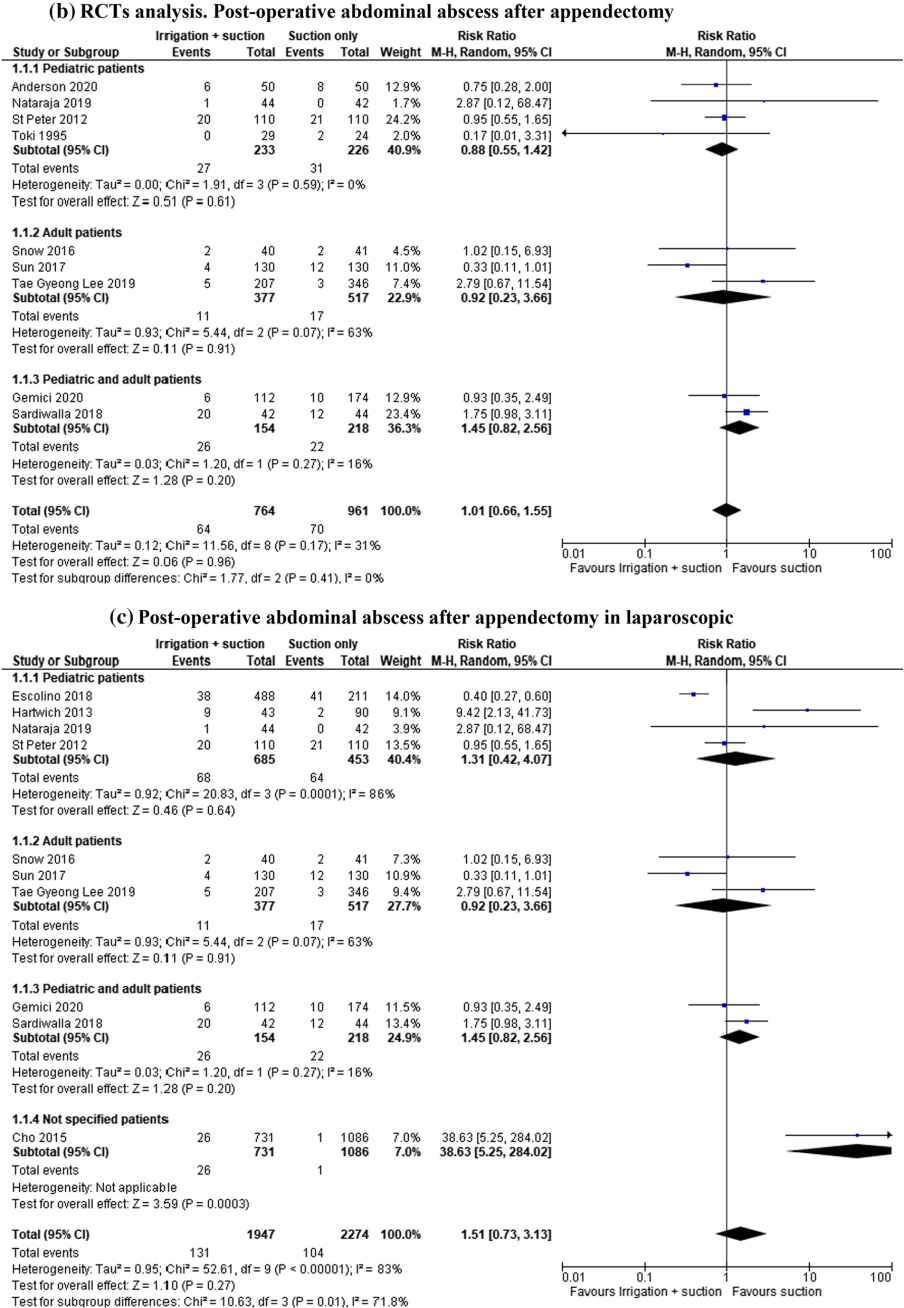
**a** Post-operative abdominal abscess after appendectomy. **b** RCT analysis. Post-operative abdominal abscess after appendectomy. **c** Post-operative abdominal abscess after appendectomy in laparoscopic group. **d** RCT analysis. Post-operative abdominal abscess after appendectomy in laparoscopic group

In the groups considering both open and laparoscopic approaches, intra-abdominal abscess was reported in 5315 patients (17 studies). The rate of this complication was lower in patients who underwent suction only, but the result was not statistically significant (RR 1.23, 95% CI 0.73–2.07; *I*^2^ = 72%) (Fig. 2a). A further analysis was performed after the exclusion of Normann’s article, published in the 1975, but the same non-statistically significant result was obtained (RR 1.27, 95% CI 0.75–2.15; *I*^2^ = 74%). Furthermore, an analysis of the RCTs (1725 patients in 9 studies) was completed: the rate of abscess was lower in patients who underwent suction only, but the result was not statistically significant although the heterogeneity in this group was lower (RR 1.01, 95% CI 0.66–1.55; *I*^2^ = 31%) (Fig. 2b).

The same outcomes were also reported in the laparoscopic approach group (4221 patients in 10 studies): the rate of abscess was lower in patients who underwent suction only, but the result was not statistically significant (RR 1.51, 95% CI 0.73–3.13; *I*^2^ = 83%) (Fig. 2c). The subgroup analysis of RCTs also showed same findings (RR 0.94, 95% CI 0.61–1.45; *I*^2^ = 83%) (Fig. 2d).

#### Reoperation for post-operative abdominal abscess after appendectomy

**Fig. 3:**
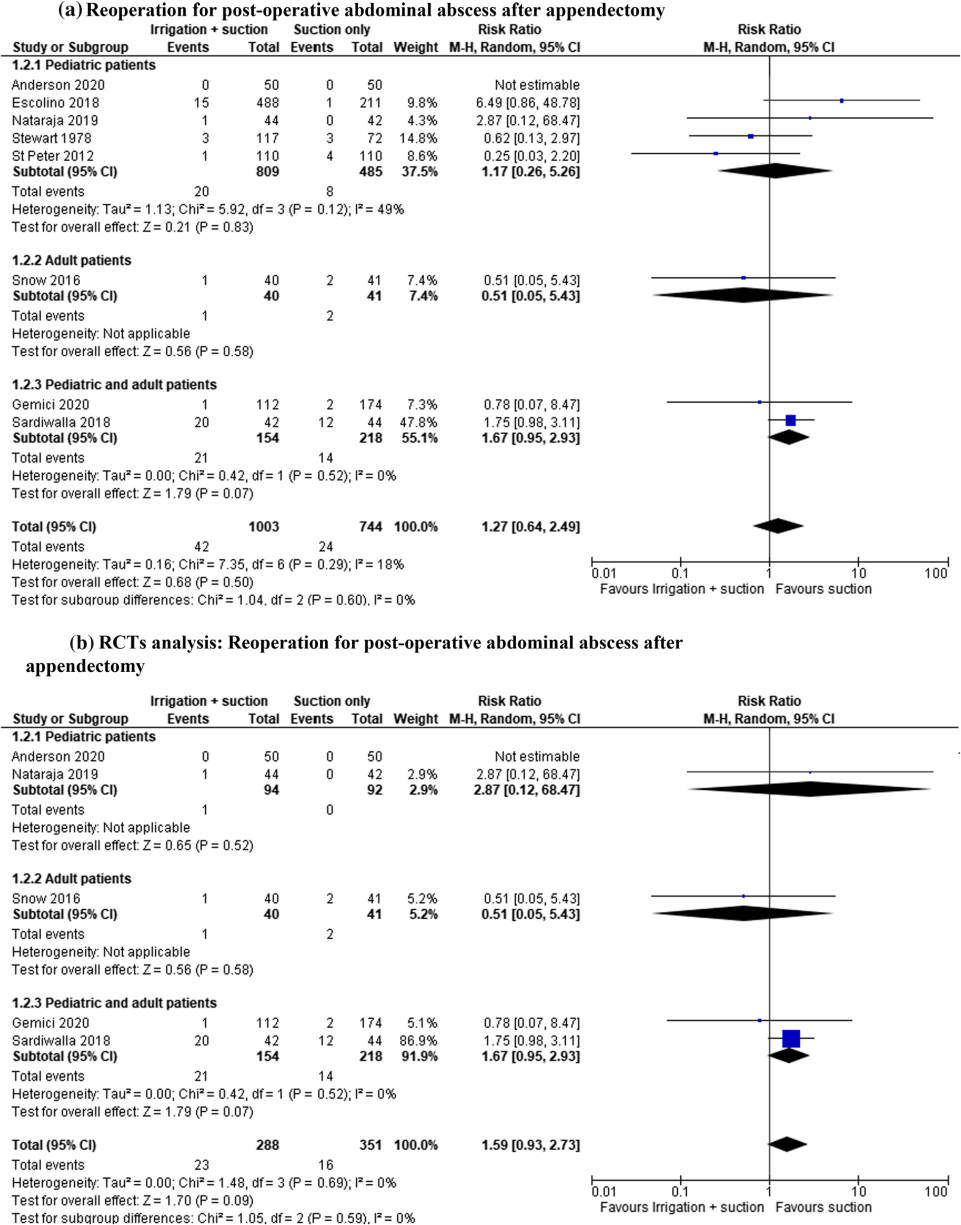

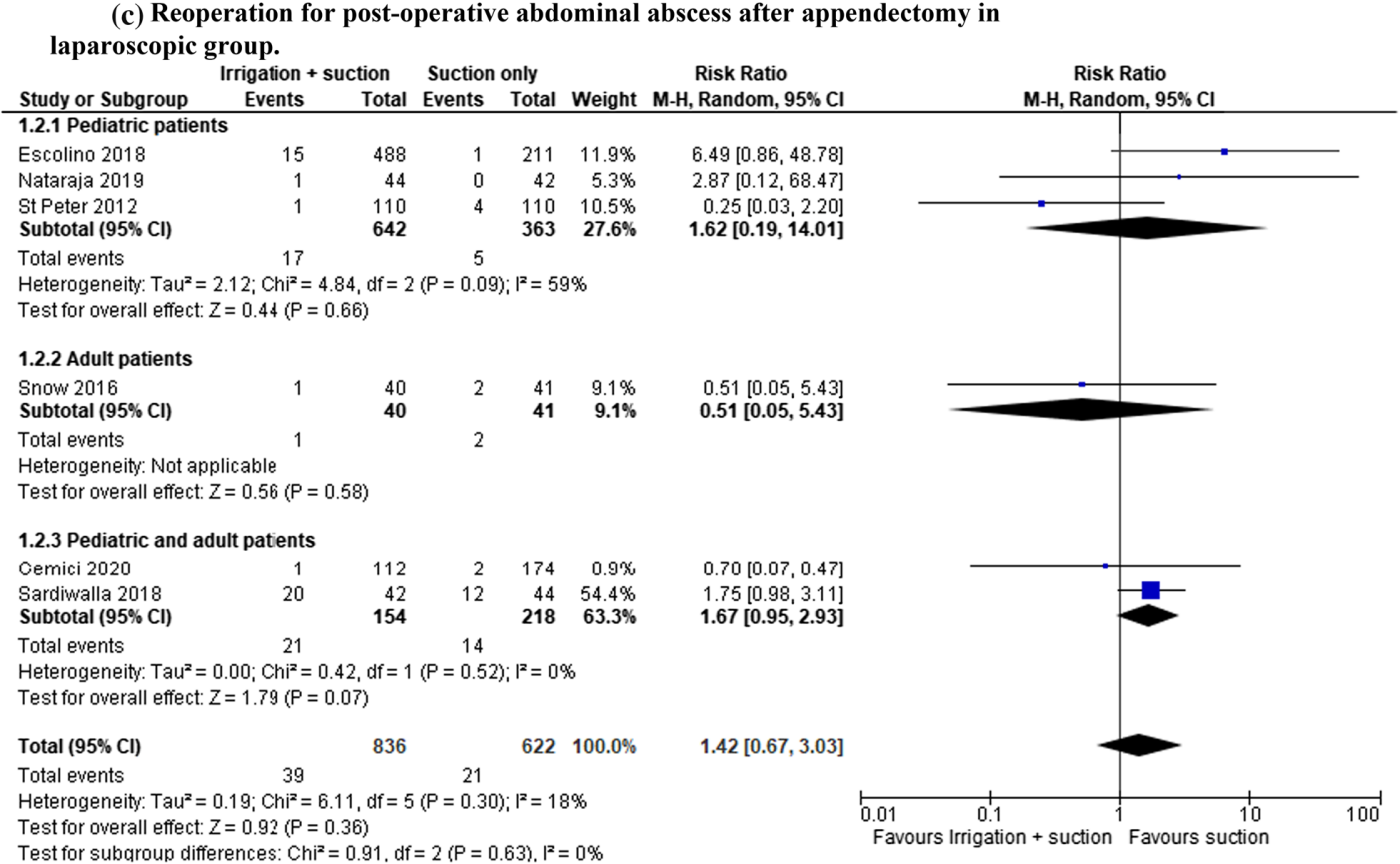
**a** Reoperation for post-operative abdominal abscess after appendectomy. **b** RCT analysis: Reoperation for post-operative abdominal abscess after appendectomy. **c** Reoperation for post-operative abdominal abscess after appendectomy in laparoscopic group

In the open and laparoscopic group, reoperation for post-operative abscess was reported in 1747 patients (8 studies) and a lower rate was registered in patients who underwent suction only; however, the results were not statistically significant (RR 1.27, 95% CI 0.04–2.49; *I*^2^ = 18%) (Fig. 3a). An analysis of the RCTs (639 patients in 5 studies) was performed. All the RCTs described a laparoscopic approach and the rate of abscess was also lower in patients who underwent suction only. The data were not statistically significant although there was no heterogeneity (RR 1.59, 95% CI 2.73–1.55; *I*^2^ = 0%) (Fig. 3b).

In the laparoscopic group, reoperation was reported in 1458 patients (six studies). The rate of this complication was also lower in patients who underwent suction only, but the result was not statistically significant (RR 1.42, 95% CI 0.64–2.49; *I*^2^ = 18%) (Fig. 3c).

### Secondary outcome

#### Operative time

**Fig. 4:**
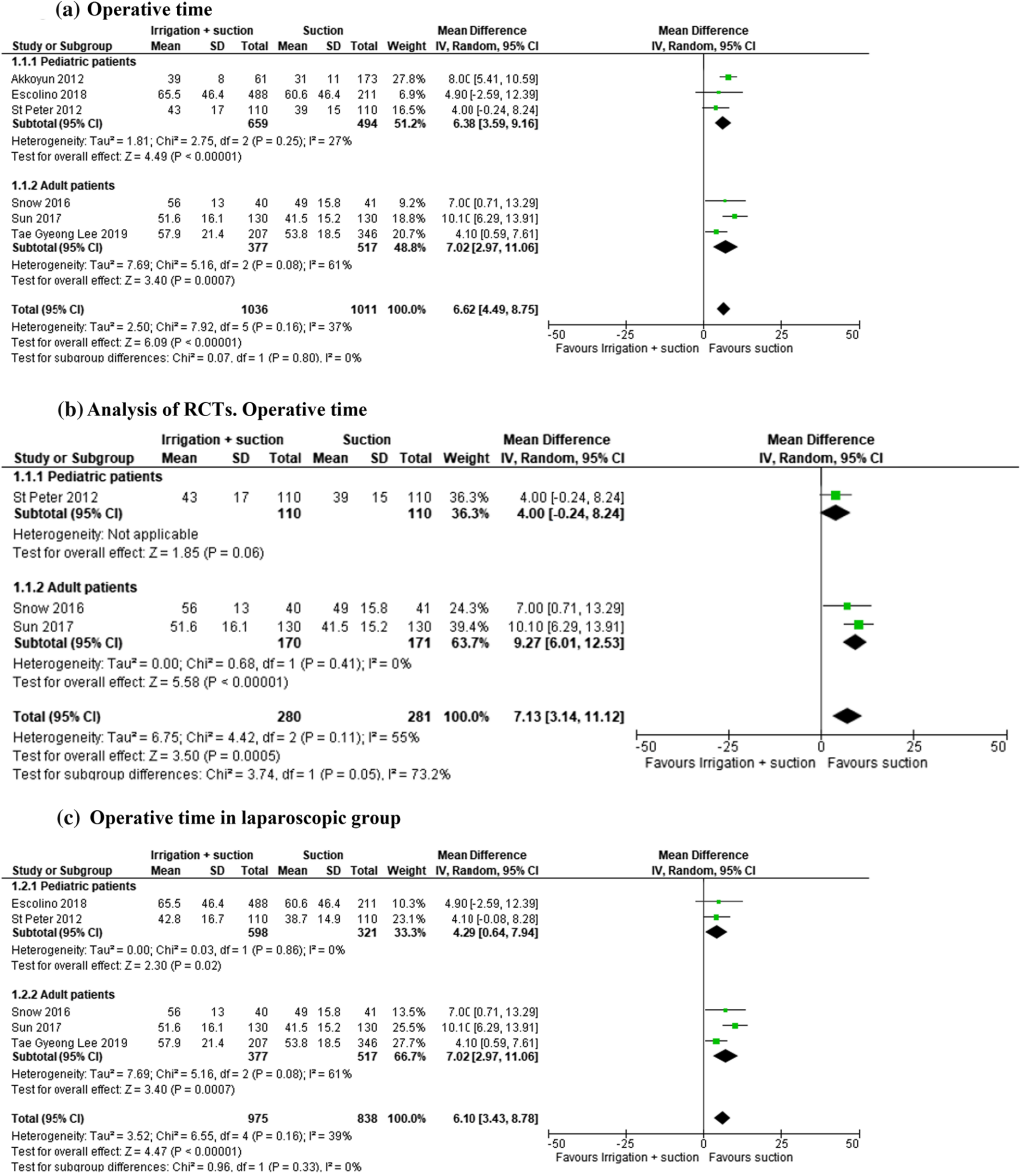
**a** Operative time. **b** Analysis of RCTs. Operative time. **c** Operative time in laparoscopic group

In the open and laparoscopic groups, operative time was evaluated in 2047 patients (6 studies) and was reported as significantly lower in patients who underwent suction only (RR 6.62, 95% CI 4.49–8.75; *I*^2^ = 37%) (Fig. 4a). The subgroup analysis of RCTs (561 patients in 3 studies) also reported a significant lower operative time in the suction group (RR 7.13, 95% CI 3.14–11.12). All patients enrolled in the RCTs underwent a laparoscopic approach (Fig. 4b).

In the laparoscopic group, this outcome was evaluated in 1813 patients (5 studies) with the operative time significantly lower in patients who underwent suction only (RR 6.10, 95% CI 3.34–8.78; *I*^2^ = 39%) (Fig. 4c).

#### Length of hospital stay

**Fig. 5:**
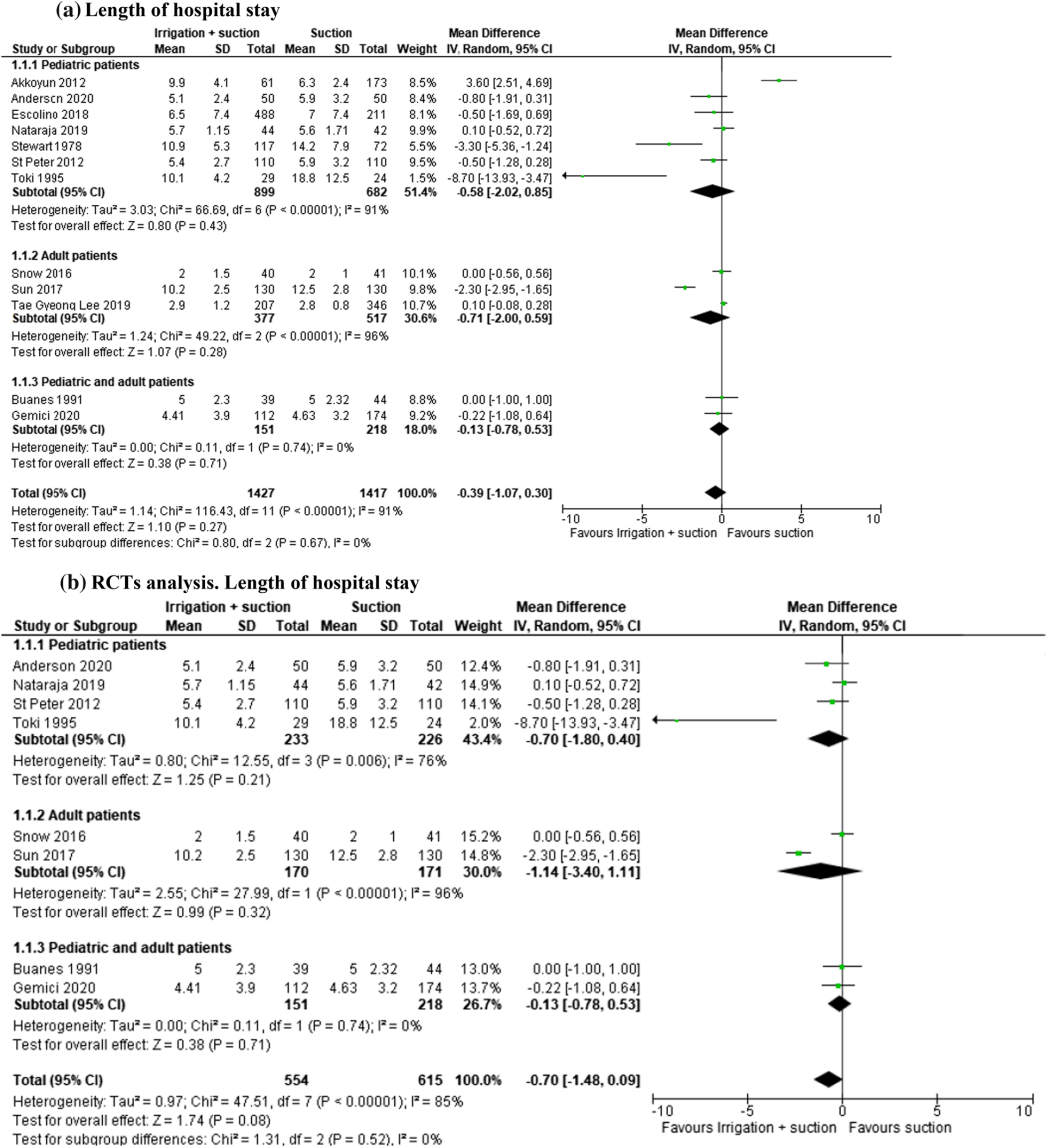

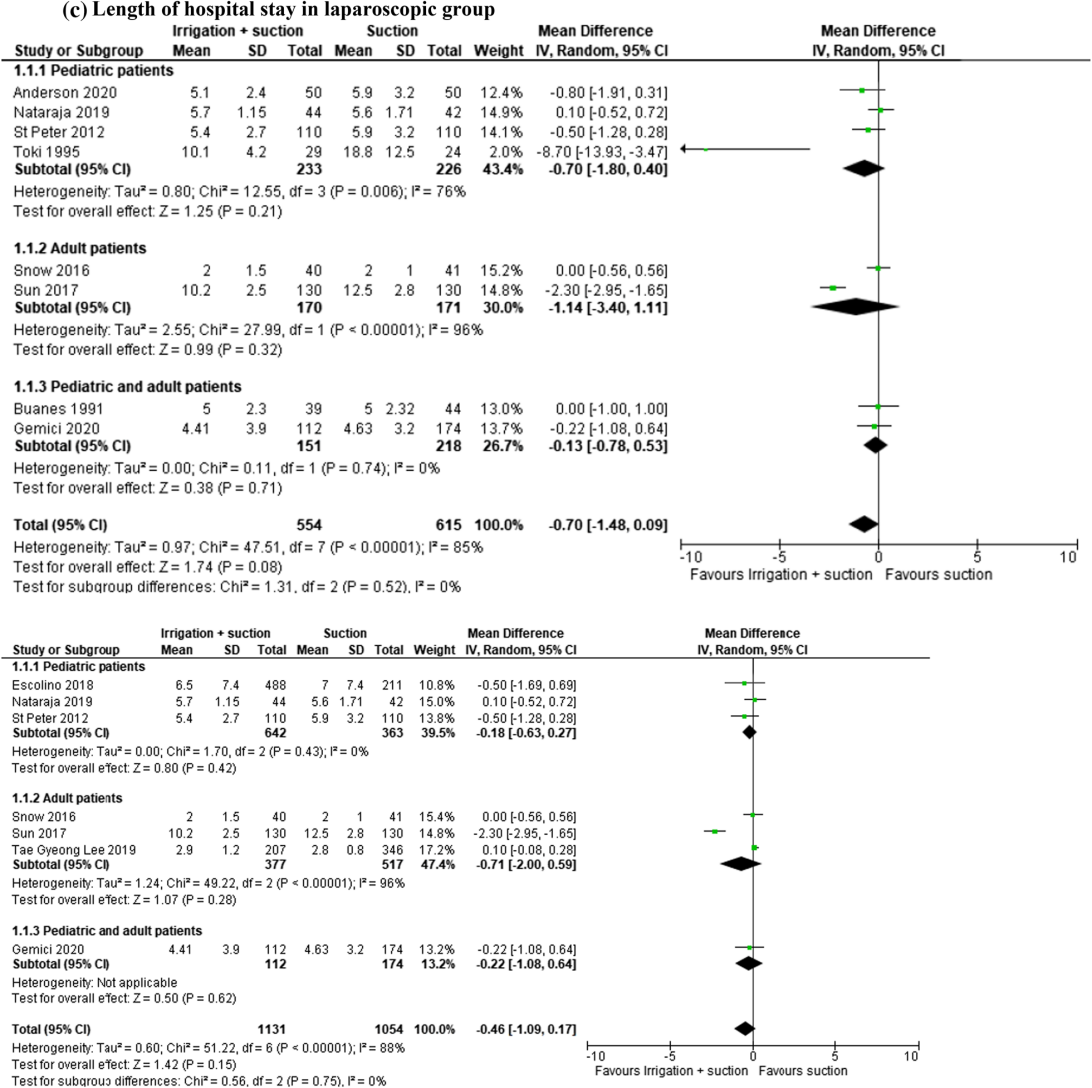

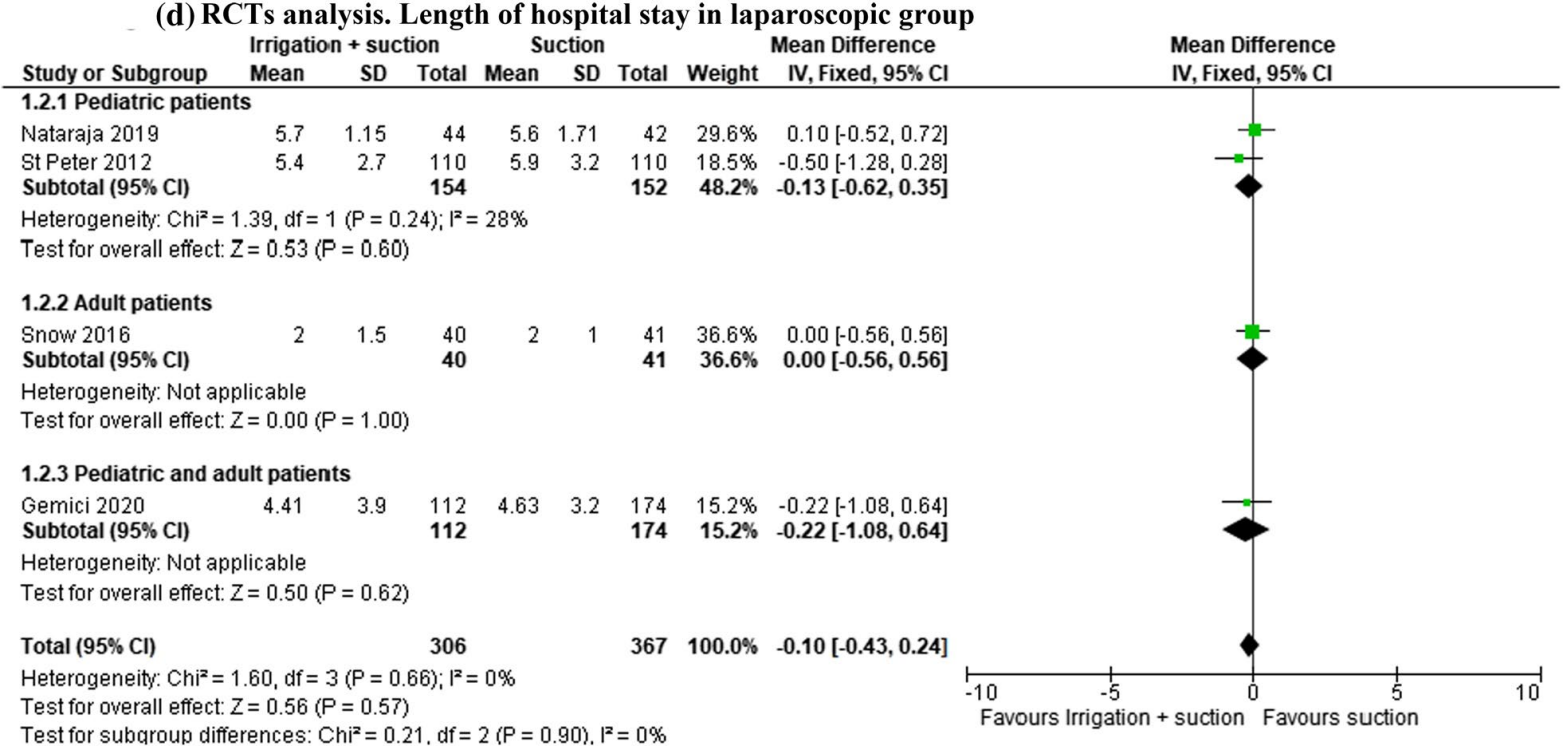
**a** Length of hospital stay. **b** RCT analysis. Length of hospital stay. **c** Length of hospital stay in laparoscopic group. **d** RCT analysis. Length of hospital stay in laparoscopic group

In the open and laparoscopic group, length of hospital stay was evaluated in 2844 patients (1^2^ studies). This outcome was lower in patients who underwent peritoneal irrigation and suction, but the result was not statistically significant (MD − 0.39, 95% CI − 1.07 to 0.30; *I*^2^ = 91%) (Fig. 5a). The same result was reported in the subgroup analysis of 1169 patients enrolled in 8 RCTs (MD − 0.70, 95% CI − 1.48 to 0.09; *I*^2^ = 85%) (Fig. 5b).

In the laparoscopic group, this outcome was studied in 2185 patients (6 studies). The length of stay was lower in patients who underwent peritoneal irrigation and suction, but the result was not statistically significant (MD − 0.46, 95% CI − 1.09 to 0.17; *I*^2^ = 88%) (Fig. 5c). The analysis of 4 RCTs (673 patients) reported the same result (MD − 0.10, 95% CI − 0.43 to 0.24; *I*^2^ = 0%) (Fig. 5d).

#### Rate of wound infections

**Fig. 6:**
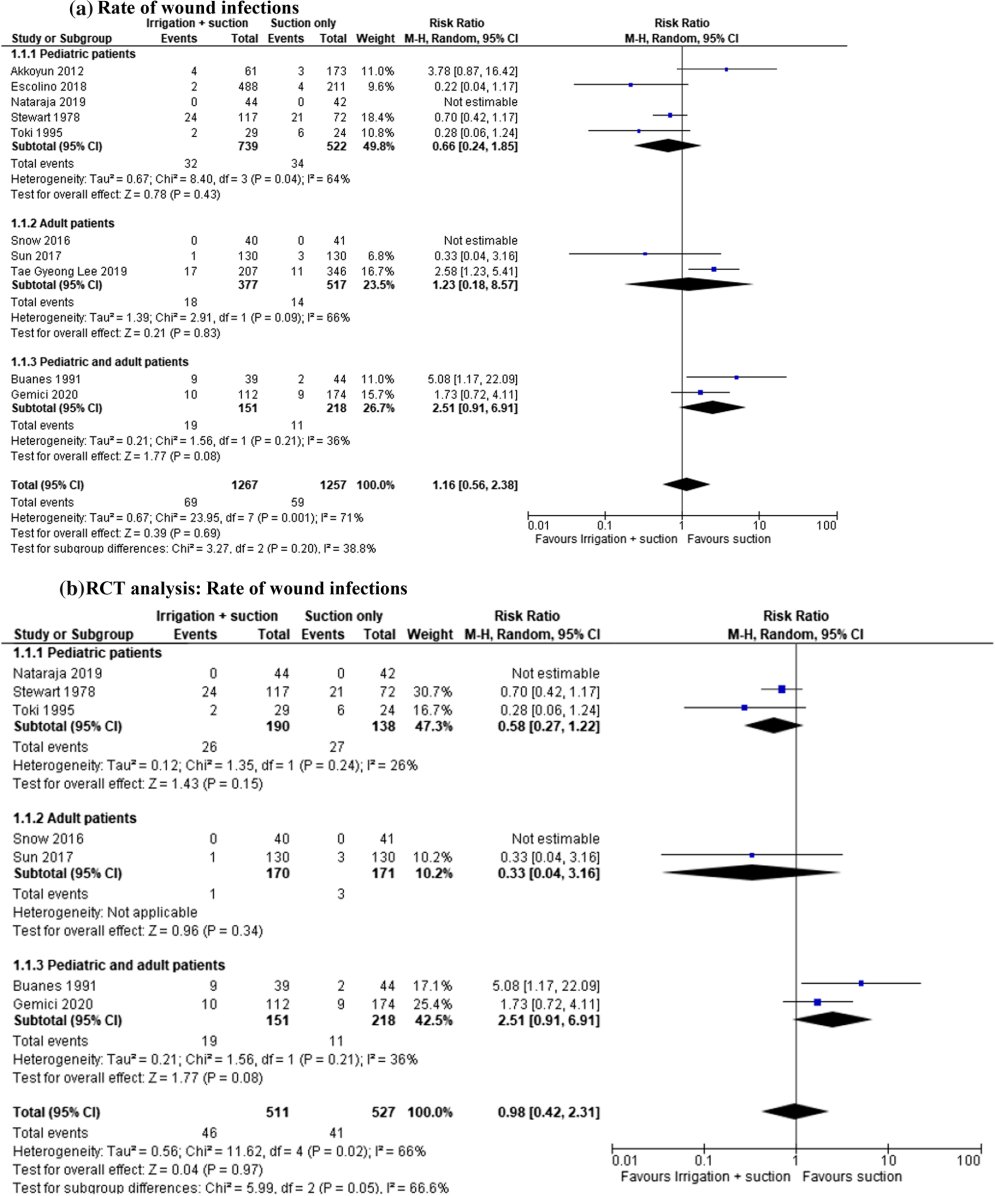

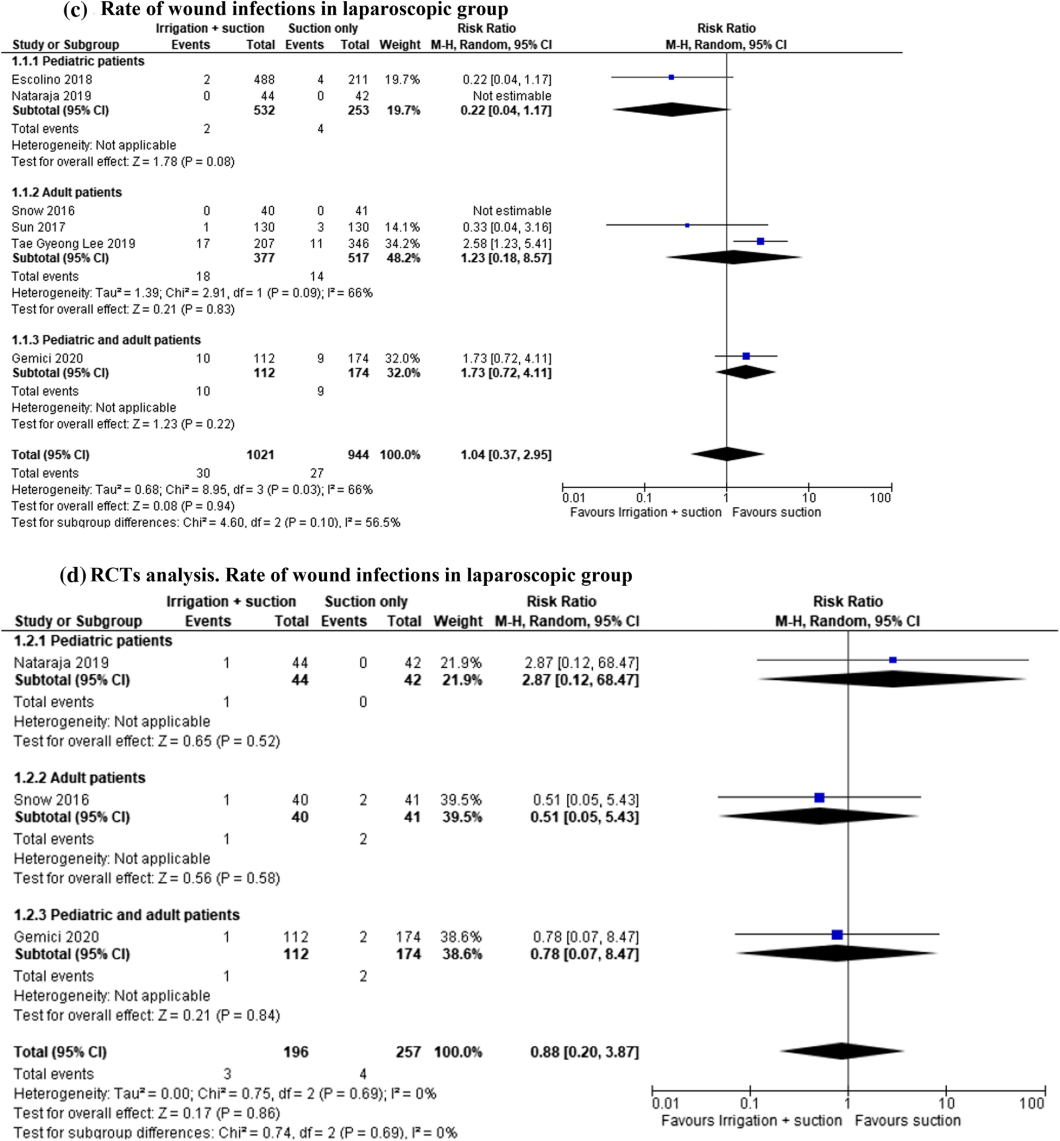
**a** Rate of wound infections. **b** RCT analysis: rate of wound infections. **c** Rate of wound infections in laparoscopic group. **d** RCT analysis. Rate of wound infections in laparoscopic group

In the open and laparoscopic group, the rates of wound infection were examined in 2524 patients (10 studies). This complication was lower in patients who underwent suction only, and the result was not statistically significant (MD 1.16, 95% CI 0.56–2.38; *I*^2^ = 71%). However, the analysis of different age groups shows contrasting results. In the paediatric group, peritoneal irrigation and suction were associated with fewer complications, while in the adult and mixed group suction only seemed to be safer (Fig. 6a). In the subgroup analysis of 1038 patients enrolled in 7 RCTs, there was no difference between the two groups (MD 0.98, 95% CI 0.42–2.31; *I*^2^ = 66%) (Fig. 6b).

In the laparoscopic group, wound infection was evaluated in 1965 patients (five studies). The rate of this complication was the same in both paediatric and adult groups (MD 1.04, 95% CI 0.37–2.95; *I*^2^ = 66%). In the subgroup analysis, there were contrasting results. In the paediatric group, peritoneal irrigation and suction seemed to be related to better outcomes, while in the adult and mixed group, suction only led to a lower complication rate (Fig. 6c). In the subgroup analysis of 453 patients enrolled in 3 RCTs, there was no difference between the two groups (MD 0.88, 95% CI 0.20–3.87; *I*^2^ = 0%) (Fig. 6d).

## Discussion

This systematic review with meta-analysis has shown similarity in terms of outcome between the use of peritoneal lavage and suction-only during appendectomy for complicated appendicitis. In particular, our review does not demonstrate a statistical difference in terms of intra-abdominal post-operative abscess, reoperation for abscess, wound infection and hospital stay, between peritoneal irrigation and suction-only of purulent material, in patient underwent appendectomy for complicated appendicitis, laparoscopically or open. Only exception was a lower operative time in suction-only group.

Acute appendicitis is one of the most common gastrointestinal-related diseases, and appendectomy represents one of the most frequently performed abdominal surgical procedures.

Unless appendectomy is considered a safe procedure, it presents an innate risk of complications.

Post-appendectomy complications reported are intra-abdominal abscess (IAA), wound infection, wound dehiscence, small bowel obstruction and bleeding. IAA occurs in 2.2% of cases after laparoscopic appendectomy. In more complicated appendicitis cases, there is a higher risk of development of a post-operative abscess [7, 10]. Perforated appendicitis was recognized as an independent risk factor for development of post-operative IAA [7–9]. Other risk factors are diabetes mellitus, young and old age [8], obesity and peritoneal irrigation [6, 8]. Despite the use of the laparoscopic approach [2], post-operative complications still present with a high incidence rate [61, 62]. The management of IAA remains controversial with different strategies suggested to decrease its incidence. Peritoneal irrigation, first described by Torek in 1903, received great emphasis as a procedure to reduce abscess occurrence. Many studies have been conducted since then, comparing peritoneal irrigation versus suction of peritoneal abscess without lavage, leading to an important heterogeneity of findings and opinions.

We performed a systematic review, according to PRISMA guidelines, comparing seventeen studies with case data for peritoneal irrigation and suction vs suction-only of the abdominal cavity during appendectomy. Analysis included the paediatric population, the adult population and combined age groups. Both laparoscopic and open procedures were included. Occurrence of intra-abdominal abscess, reoperation for abscess, operation time, length of stay and wound infections were assessed.

Analysis of the literature revealed early studies which seemed to show the efficiency of peritoneal irrigation in reducing post-operative abscess [10], especially in adult populations. The success of irrigation was thought to be related to bacterial load dilution. More recent studies suggest the ineffectiveness of irrigation in reducing post-operative IAA [6, 8, 10] as the effect of bacterial load dilution appears to be temporary. Some current evidence indeed suggests that peritoneal irrigation may increase the incidence of abscess formation in perforated appendicitis [10, 55].

In 2013, St. Peter defined three mechanisms potentially responsible for the ineffectiveness of peritoneal irrigation:

1. bacteria adhere to the peritoneal mesothelial cells, so irrigations do not decrease the microorganism load on the peritoneum;
2. irrigation may cause diffuse or remote bacterial inoculation, thus spreading the pollution [8];
3. irrigation may dilute mediators of phagocytosis as opsonic proteins and immunoglobulins.

Nevertheless, many surgeons continue to employ peritoneal lavage during appendectomy [63].

A minority of authors only found that peritoneal irrigation could have some benefit in reducing post-operative IAA incidence [51, 60]. Some authors found that peritoneal irrigation seems to increase the risk of developing post-operative IAA after appendectomy, independently from a laparoscopic or open approach [6, 8].

Some authors reported differences between irrigation and suction and suction only, especially in children, in favour of suction only [10, 38]. One study was interrupted due to an excess risk of developing an abscess in the lavage group compared to suction-only group [55].

Most of the analysed studies did not find statistical differences between suction-only versus peritoneal irrigation and suction to prevent post-operative IAA after appendectomy for perforated appendicitis [8].

Many articles have been published around the use of peritoneal irrigation and suction versus suction only during appendectomy. In this wide and heterogeneous panorama, no articles succeeded in defining whether irrigation is an effective method to reduce post-operative IAA. Only a few studies suggested that peritoneal irrigation could have some benefit in reducing the incidence of post-operative IAA [51, 60], and the other papers reported that peritoneal irrigation seemed to increase the risk of post-operative IAA after appendectomy, regardless of a laparoscopic or open procedure [6, 8]. Some authors also reported a prolonged operation time in the lavage group.

In this systematic review, we analysed 17 selected studies (9 RCTs and 8 CCTs) with 5315 patients (2532 irrigation and suction vs 2783 irrigation).

We have identified primary (post-operative IAA and reoperation) and secondary outcomes (operative time, length of hospitalization, wound infections).

Regarding primary outcome, in both laparoscopic and open appendectomy, in children and adult population, we found no statistical difference in terms of onset of intra-abdominal abscess, although the rate of this complication appeared to be lower in patients who underwent suction only.

Same results for reoperation for post-operative abscess: a lower rate was registered in patients who underwent suction only; however, the results were not statistically significant, in both open and laparoscopic groups.

For secondary outcome, in the open and laparoscopic groups, operative time was reported as significantly lower in patients who underwent suction only.

In the open and laparoscopic group, length of hospital stay was lower in patients who underwent peritoneal irrigation and suction, but the result was not statistically significant.

In the open and laparoscopic group, the rates of wound infection were lower in patients who underwent suction only, although the result was not statistically significant. However, the analysis of different age groups shows contrasting results. In the paediatric group, peritoneal irrigation and suction were associated with fewer complications, while in the adult and mixed group suction only seemed to be safer.

Unfortunately, these publications are not homogeneous as some of them analyse a laparoscopic approach only, other just open procedure or other both of them. The population studied were also different: some articles analyse paediatric population, instead other adult population or both.

The risk of bias in the RCTs was assessed using methods described in the Cochrane Handbook for Systematic Reviews of Interventions and the Risk of Bias In Non-randomized Studies of Interventions (ROBINS-I) assessment tool.

The risk of bias was reported as low to moderate.

According to ROBINS-I tool, three studies were assessed as low risk of overall bias, while five were determined to have a moderate risk. Regarding bias due to confounding, four studies were evaluated as having a low risk, four studies seemed to have a moderate risk of bias due to confounding. Regarding bias in classification of the interventions, all studies had a low risk. Outcomes were clearly defined and measures were not influenced by knowledge of the intervention received. The methods of outcome assessment were comparable across intervention groups, and no systematic errors were detected. Analysing bias due to missing data, three reviews were assessed as moderate risk.

This systematic review and meta-analysis has failed to demonstrate the superiority of employing intra-operative peritoneal irrigation and suction over suction-only to reduce the rate of post-operative intra-abdominal abscess after appendectomy (RR 1.23, 95% CI 0.73–2.07; *I*^2^ = 72%). Furthermore, the absolute number of abscesses was fewer in suction-only groups both in the overall procedure group (6.91%, 175/2532, in lavage group vs 4.67%, 130/2783, in suction group) and in laparoscopic procedures (6.73%, 131/1947, in lavage group vs 4.57%, 104/2274, in suction group). The subgroup analysis based on patient age yielded the same results.

Operation time (RR 6.10, 95% CI 3.34–8.78; *I*^2^ = 39%) and rate of reoperation (RR 6.62, 95% CI 4.49–8.75; *I*^2^ = 37%) were significantly lower in patients who underwent suction only.

In the paediatric populations, the lavage groups showed some advantage with regard to the incidence of wound infection for laparoscopic appendectomy, although this was not statistically significant (MD 1.16, 95% CI 0.56–2.38; *I*^2^ = 71%).

Overall no statistical significance was found in the defined primary and secondary outcomes, except for operation time, between peritoneal irrigation and suction group compared to suction only in treating complicated appendicitis. Clinical advantages, which were not statistically significant, for reduced incidence of post-operative intra-abdominal abscess and reoperation for abscess, operative time and wound infection were detected in the suction-only groups.

## Conclusion

In conclusion, our analysis did not find a statistical advantage in terms of major outcome in the use of intra-operative peritoneal irrigation on the avoidance of IAA, neither were disadvantages detected. Nevertheless, many articles reported clinical evidence of benefit in the suction-only groups for treating complicated appendicitis in reducing post-operative risk of abscess, wound infection and operative times. This meta-analysis suggests the use of an only-suction approach of purulent liquid in cases of localized peritonitis during appendectomy. In the presence of diffuse purulent peritonitis, a prolonged lavage until the abdomen is completed cleansed may still be preferable.

## Supplementary Information

**Additional file 1**. SDC searches.

**Additional file 2:Table S1.** Excluded studies.

**Additional file 3:** SDC baseline.

**Additional file 4:** SDC 3a: Risk-of-bias graph: review authors' judgments about each risk–of-bias item presented as percentages across all included studies.

**Additional file 5:** SDC 3b: Risk-of-bias summary: review authors' judgments about each risk-of-bias item for includedstudies.

**Additional file 6:** SDC 4: Risk of bias domains.

## Data Availability

The literature was searched using PubMed, Scopus and Web of Science. Code availability: PROSPERO international prospective register of systematic reviews, Number CRD42020186848.

## Abbreviations

RCT: Randomized, controlled trial
CCT: Controlled clinical trials
PRISMA: Preferred Reporting Items for Systematic Reviews and Meta-analyses
IAA: Intra-abdominal abscess
WSES: World Society of Emergency Surgery
BMI: Body mass index
RR: Risk ratios
