# Supplementary material for: Aspiration versus peritoneal lavage in appendicitis: a meta-analysis
Source: World J Emerg Surg. 2021 Sep 6;16:44. doi: 10.1186/s13017-021-00391-y (PMC8419906; doi:10.1186/s13017-021-00391-y)
Supplement: Supplementary file 1 — Additional file 1. SDC searches. [file 13017_2021_391_MOESM1_ESM.docx]

In the meta-analysis “Aspiration vs. Peritoneal lavage in appendicitis: a meta-analysis”, the search details were the following:

Search details on PubMed

"peritoneal irrigation"[All Fields] AND ("appendicitis"[MeSH Terms] OR "appendicitis"[All Fields] OR ("ruptured"[All Fields] AND "appendicitis"[All Fields]) OR "ruptured appendicitis"[All Fields])

Results: 22 articles

"peritoneal lavage"[All Fields] AND ("appendicitis"[MeSH Terms] OR "appendicitis"[All Fields] OR ("ruptured"[All Fields] AND "appendicitis"[All Fields]) OR "ruptured appendicitis"[All Fields])

Results: 106 articles

Search details on Scopus

ALL ( peritoneal AND lavage AND appendicitis )

Results: 539 articles

ALL ( peritoneal AND irrigation AND appendicitis )

Results: 71 articles

Search details on WOS

TOPIC: (peritoneal  AND lavage  AND appendicitis)

Results: 41 articles

TOPIC: (peritoneal  AND irrigation  AND appendicitis)

Results: 30 articles
