# Supplementary material for: Aspiration versus peritoneal lavage in appendicitis: a meta-analysis
Source: World J Emerg Surg. 2021 Sep 6;16:44. doi: 10.1186/s13017-021-00391-y (PMC8419906; doi:10.1186/s13017-021-00391-y)
Supplement: Supplementary file 2 — Additional file 2:Table S1. Excluded studies. [file 13017_2021_391_MOESM2_ESM.docx]

**Table S1: Excluded studies**

| Authors | Reason for exclusion |
| --- | --- |
| Siotos 2019 | Meta-analysis |
| Bi 2019 | Meta-analysis |
| Gammeri 2018 | Meta-analysis |
| Hajibandeh 2018 | Meta-analysis |
| Bölke 2002 | Continuous post-operative lavage |
| Schwarz 2007 | Continuous post-operative lavage |
| Schneider 2005 | Use of taurolidine lavage |
| Gruel 1985 | Not comparative studies |
| Udén 1983 | Continuous post-operative lavage |
| Tighe 1982 | Use of betadine |
| Gjessing 1976 | Continuous post-operative lavage |
