# Supplementary material for: Aspiration versus peritoneal lavage in appendicitis: a meta-analysis
Source: World J Emerg Surg. 2021 Sep 6;16:44. doi: 10.1186/s13017-021-00391-y (PMC8419906; doi:10.1186/s13017-021-00391-y)
Supplement: Supplementary file 3 — Additional file 3: SDC baseline. [file 13017_2021_391_MOESM3_ESM.docx]

|  | **Normann, 1975** | | **Stewart, 1978** | | **Buanes, 1991** | | **Toki, 1995** | | **Moore, 2011** | |
| --- | --- | --- | --- | --- | --- | --- | --- | --- | --- | --- |
|  | ***Irrigation*** | ***Suction*** | ***Irrigation*** | ***Suction*** | ***Irrigation*** | ***Suction*** | ***Irrigation*** | ***Suction*** | ***Irrigation*** | ***Suction*** |
| **Age** | 5-88 (46) | 4-89 (44) | N/S | N/S | 6-80 (39) | 6-82 (41) | N/S | N/S | 41,7 (± 17,5) | 41,9 (± 16,1) |
| **Male sex** | N/S | N/S | N/S | N/S | 18 | 20 | N/S | N/S | N/S | N/S |
| **BMI** | N/S | N/S | N/S | N/S | N/S | N/S | N/S | N/S | N/S | N/S |
| **ASA** | N/S | N/S | N/S | N/S | N/S | N/S | N/S | N/S | N/S | N/S |
| **Duration of abdominal pain** | N/S | N/S | N/S | N/S | 42 h | 42 h | N/S | N/S | 2,5 (± 5,5) days | 2,6 (± 6,5) |
| **Preoperative fever** | N/S | N/S | N/S | N/S | N/S | N/S | N/S | N/S | N/S | N/S |
| **Retrocecal type appendix** | N/S | N/S | N/S | N/S | N/S | N/S | N/S | N/S | N/S | N/S |
| **White blood cell** | N/S | N/S | N/S | N/S | N/S | N/S | N/S | N/S | N/S | N/S |
| **Neutrophil percentage** | N/S | N/S | N/S | N/S | N/S | N/S | N/S | N/S | N/S | N/S |
| **Hemoglobin (g/dL)** | N/S | N/S | N/S | N/S | N/S | N/S | N/S | N/S | N/S | N/S |
| **C-reactive protein (mg/dL)** | N/S | N/S | N/S | N/S | N/S | N/S | N/S | N/S | N/S | N/S |
| **Total bilirubin (mg/dL)** | N/S | N/S | N/S | N/S | N/S | N/S | N/S | N/S | N/S | N/S |
| **Albumin (g/dL)** | N/S | N/S | N/S | N/S | N/S | N/S | N/S | N/S | N/S | N/S |

|  | **Akkoyun, 2012** | | **St. Peter, 2012** | | **Hartwich, 2013** | | **Cho, 2015** | |
| --- | --- | --- | --- | --- | --- | --- | --- | --- |
|  | ***Irrigation*** | ***Suction*** | ***Irrigation*** | ***Suction*** | ***Irrigation*** | ***Suction*** | ***Irrigation*** | ***Suction*** |
| **Age** | 8,8 (± 3,7) | 9,1 (± 4,0) | 10,4 (±3,8) | 9,7 (± 3,6) | 10,2 (±4,2) | 10,4 (±3,5) | N/S | N/S |
| **Male sex** | N/S | N/S | 52,70% | 59,10% | 77 (55%) | 49 (50%) | N/S | N/S |
| **BMI** | N/S | N/S | 60,7 (±31,9) | 65,0 (±32,3) | N/S | N/S | N/S | N/S |
| **ASA** | N/S | N/S | N/S | N/S | N/S | N/S | N/S | N/S |
| **Duration of abdominal pain** | N/S | N/S | 3,1 (±2,0) days | 3,1 (±2,1) | 49,1 (± 33,0) h | 48,8 (±32,7) | N/S | N/S |
| **Preoperative fever** | N/S | N/S | 37,8 (± 0,9) °C | 31,8 (±1,0) °C | N/S | N/S | N/S | N/S |
| **Retrocecal type appendix** | N/S | N/S | N/S | N/S | N/S | N/S | N/S | N/S |
| **White blood cell** | 15,7 (± 4,1) (kg/mm³) | 16,6 (± 4,5) | 17,3 (± 5,0) (1000cells/mm³) | 17,1 (± 5,9) | 16,06 (± 5,7) (10⁹cells/L) | 17,19 (± 4,7) | N/S | N/S |
| **Neutrophil percentage** | N/S | N/S | N/S | N/S | N/S | N/S | N/S | N/S |
| **Hemoglobin (g/dL)** | N/S | N/S | N/S | N/S | N/S | N/S | N/S | N/S |
| **C-reactive protein (mg/dL)** | N/S | N/S | N/S | N/S | N/S | N/S | N/S | N/S |
| **Total bilirubin (mg/dL)** | N/S | N/S | N/S | N/S | N/S | N/S | N/S | N/S |
| **Albumin (g/dL)** | N/S | N/S | N/S | N/S | N/S | N/S | N/S | N/S |

|  | **Snow, 2016** | | **Sun, 2017** | | **Sardiwalla, 2018** | | **Escolino, 2018** | |
| --- | --- | --- | --- | --- | --- | --- | --- | --- |
|  | ***Irrigation*** | ***Suction*** | ***Irrigation*** | ***Suction*** | ***Irrigation*** | ***Suction*** | ***Irrigation*** | ***Suction*** |
| **Age** | 32 (22,5-39) | 20 (20-38) | 37,9 (± 19,1) | 38,7 (±18,5) | 25,7 (±17,01) | 27,4 (±11,88) | 10,2 | 10,9 |
| **Male sex** | 27 | 25 | 73 | 71 | 19 (45,2%) | 29 (65,9%) | 340 | 120 |
| **BMI** | N/S | N/S | 24,1 (±3,1) | 23,8 (±2,7) | N/S | N/S | N/S | N/S |
| **ASA** | 1 (1-2) | 1 (1-2) | N/S | N/S | N/S | N/S | N/S | N/S |
| **Duration of abdominal pain** | N/S | N/S | N/S | N/S | N/S | N/S | N/S | N/S |
| **Preoperative fever** | 37,0 (36,7-37,3) °C | 36,9 (36,6-37,4) | N/S | N/S | 37,3 (±0,46) °C | 37,4 (±0,60) | N/S | N/S |
| **Retrocecal type appendix** | N/S | N/S | N/S | N/S | N/S | N/S | N/S | N/S |
| **White blood cell** | 14,5 (12,4-17,7) (X1000/mm³) | 15,9 (12,8-18,4) | 16,9 (±2,6) (X1000/mm³) | 16,7 (±2,9) | 13,7 (±1,44) | 13,7 (±1,88) | 15,1 | 14,85 |
| **Neutrophil percentage** | N/S | N/S | N/S | N/S | N/S | N/S | N/S | N/S |
| **Hemoglobin (g/dL)** | N/S | N/S | N/S | N/S | N/S | N/S | N/S | N/S |
| **C-reactive protein (mg/dL)** | N/S | N/S | N/S | N/S | N/S | N/S | 12,8 | 12,5 |
| **Total bilirubin (mg/dL)** | N/S | N/S | N/S | N/S | N/S | N/S | N/S | N/S |
| **Albumin (g/dL)** | N/S | N/S | N/S | N/S | N/S | N/S | N/S | N/S |

|  | **Tae Gyeong Lee, 2019** | | **Nataraja, 2019** | | **Gemici, 2020** | | **Anderson, 2020** | |
| --- | --- | --- | --- | --- | --- | --- | --- | --- |
|  | ***Irrigation*** | ***Suction*** | ***Irrigation*** | ***Suction*** | ***Irrigation*** | ***Suction*** | ***Irrigation*** | ***Suction*** |
| **Age** | 40,6 (±15,6) | 39,8 (±15,5) | 9,5 (3-16,0) | 10 (4-16,0) | 36,22 (± 18,6) | 34,47 (± 17,40) | 10,4 (± 3,6) | 10,6 (± 4,0) |
| **Male sex** | 111 (53,6) | 160 (46,2) | 21 | 19 | 73 (65,2%) | 114 (65,5%) | 32 (64%) | 28 (56%) |
| **BMI** | 23,5 (±3,4) | 23,1 (± 3,2) | N/S | N/S | N/S | N/S | 19,7 (± 5,0) | 21,5 (± 6,4) |
| **ASA** | 6 (2,9) (ASA>3) | 12 (3,5) | N/S | N/S | N/S | N/S | N/S | N/S |
| **Duration of abdominal pain** | 1,5 (±0,8) | 1,6 (± 1,1) | 48 (12-336) h | 48 (7-120) | N/S | N/S | 42 (24-72) h | 48 (24-96) |
| **Preoperative fever** | N/S | N/S | N/S | N/S | N/S | N/S | N/S | N/S |
| **Retrocecal type appendix** | 56 | 71 | N/S | N/S | N/S | N/S | N/S | N/S |
| **White blood cell** | 12,8 (±4,3) (X10³cells/μL) | 12,4 (± 3,9) | N/S | N/S | N/S | N/S | 17,2 ± 4,9 | 17,7 ± 5,5 |
| **Neutrophil percentage** | 75,6 (±10,8) | 76,0 (±10,8) | N/S | N/S | N/S | N/S | N/S | N/S |
| **Hemoglobin (g/dL)** | 14,5 (±3,6) | 14,1 (± 1,5) | N/S | N/S | N/S | N/S | N/S | N/S |
| **C-reactive protein (mg/dL)** | 2,3 (±3,2) | 2,4 (±3,4) | N/S | N/S | N/S | N/S | N/S | N/S |
| **Total bilirubin (mg/dL)** | 1,2 (±0,5) | 1,2( ±0,5) | N/S | N/S | N/S | N/S | N/S | N/S |
| **Albumin (g/dL)** | 4,1 (±0,3) | 4,2(± 0,3) | N/S | N/S | N/S | N/S | N/S | N/S |
