# Supplementary material for: Aspiration versus peritoneal lavage in appendicitis: a meta-analysis
Source: World J Emerg Surg. 2021 Sep 6;16:44. doi: 10.1186/s13017-021-00391-y (PMC8419906; doi:10.1186/s13017-021-00391-y)

SDC 3a: Risk-of-bias graph: review authors' judgments about each risk–of-bias item presented as percentages across all included studies.


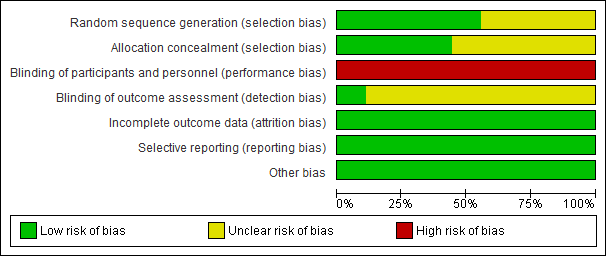

Supplement: Supplementary file 4 — Additional file 4: SDC 3a: Risk-of-bias graph: review authors' judgments about each risk–of-bias item presented as percentages across all included studies. [file 13017_2021_391_MOESM4_ESM.docx]
