# Supplementary material for: Aspiration versus peritoneal lavage in appendicitis: a meta-analysis
Source: World J Emerg Surg. 2021 Sep 6;16:44. doi: 10.1186/s13017-021-00391-y (PMC8419906; doi:10.1186/s13017-021-00391-y)

SDC 3b: Risk-of-bias summary: review authors' judgments about each risk-of-bias item for included studies.


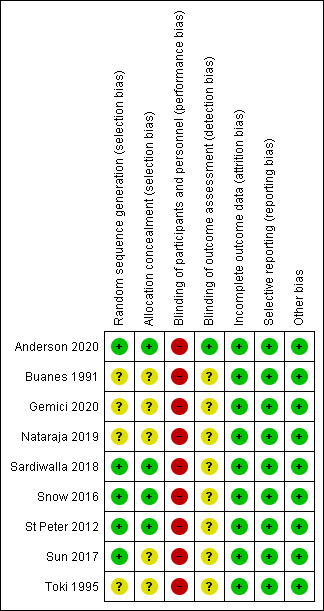

Supplement: Supplementary file 5 — Additional file 5: SDC 3b: Risk-of-bias summary: review authors' judgments about each risk-of-bias item for includedstudies. [file 13017_2021_391_MOESM5_ESM.docx]
