# Supplementary figures and images for: Aspiration versus peritoneal lavage in appendicitis: a meta-analysis
Source: World J Emerg Surg. 2021 Sep 6;16:44. doi: 10.1186/s13017-021-00391-y (PMC8419906; doi:10.1186/s13017-021-00391-y)

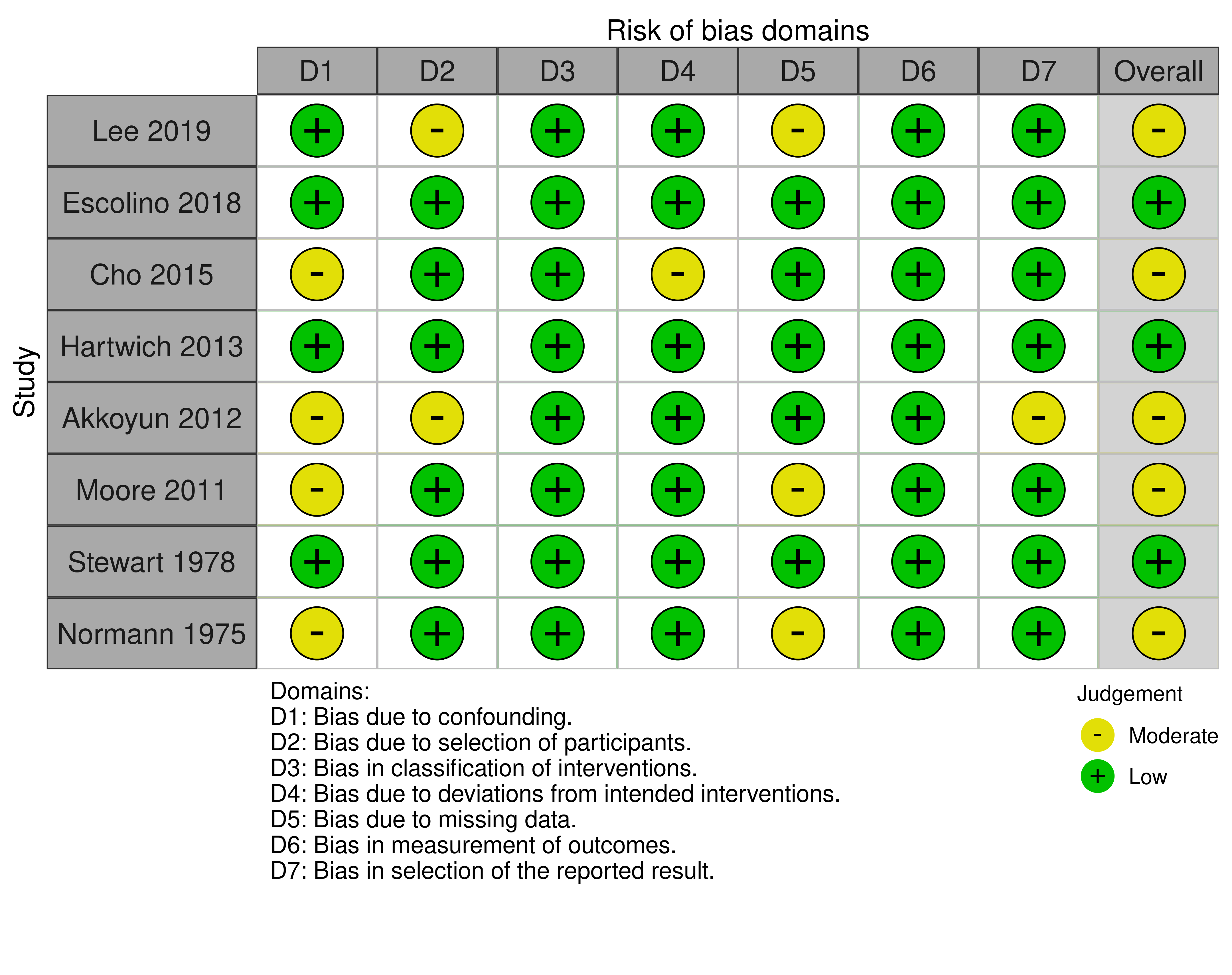

Supplement: Supplementary file 6 — Additional file 6: SDC 4: Risk of bias domains. [file 13017_2021_391_MOESM6_ESM.jpeg]
